# α-Bisabolol, a Dietary Sesquiterpene, Attenuates Doxorubicin-Induced Acute Cardiotoxicity in Rats by Inhibiting Cellular Signaling Pathways, Nrf2/Keap-1/HO-1, Akt/mTOR/GSK-3β, NF-κB/p38/MAPK, and NLRP3 Inflammasomes Regulating Oxidative Stress and Inflammatory Cascades

**DOI:** 10.3390/ijms241814013

**Published:** 2023-09-13

**Authors:** Mohamed Fizur Nagoor Meeran, Seenipandi Arunachalam, Sheikh Azimullah, Dhanya Saraswathiamma, Alia Albawardi, Saeeda Almarzooqi, Niraj Kumar Jha, Sandeep Subramanya, Rami Beiram, Shreesh Ojha

**Affiliations:** 1 Department of Pharmacology and Therapeutics, College of Medicine and Health Sciences, United Arab Emirates University, Al Ain P.O. Box 15551, United Arab Emirates; nagoormeeran1985@uaeu.ac.ae (M.F.N.M.); rbeiram@uaeu.ac.ae (R.B.); 2 Department of Pathology, College of Medicine and Health Sciences, United Arab Emirates University, Al Ain P.O. Box 15551, United Arab Emirates; 3 Department of Biotechnology, School of Engineering and Technology (SET), Sharda University, Greater Noida 201310, Uttar Pradesh, India; 4 Department of Physiology, College of Medicine and Health Sciences, United Arab Emirates University, Al Ain P.O. Box 15551, United Arab Emirates; 5 Zayed Bin Sultan Center for Health Sciences, United Arab Emirates University, Al Ain P.O. Box 15551, United Arab Emirates

**Keywords:** α-Bisabolol, doxorubicin, Nrf2/Keap-1/HO-1 pathway, Akt/mTOR/GSK-3β signaling, NF-κB/p38/MAPK activation and NLRP3 inflammasome

## Abstract

Cancer chemotherapy with doxorubicin (DOX) may have multiorgan toxicities including cardiotoxicity, and this is one of the major limitations of its clinical use. The present study aimed to evaluate the cardioprotective role of α-Bisabolol (BSB) in DOX-induced acute cardiotoxicity in rats and the underlying pharmacological and molecular mechanisms. DOX (12.5 mg/kg, single dose) was injected intraperitoneally into the rats for induction of acute cardiotoxicity. BSB was given orally to rats (25 mg/kg, p.o. twice daily) for a duration of five days. DOX administration induced cardiac dysfunction as evidenced by altered body weight, hemodynamics, and release of cardio-specific diagnostic markers. The occurrence of oxidative stress was evidenced by a significant decline in antioxidant defense along with a rise in lipid peroxidation and hyperlipidemia. Additionally, DOX also increased the levels and expression of proinflammatory cytokines and inflammatory mediators, as well as activated NF-κB/MAPK signaling in the heart, following alterations in the Nrf2/Keap-1/HO-1 and Akt/mTOR/GSK-3β signaling. DOX also perturbed NLRP3 inflammasome activation-mediated pyroptosis in the myocardium of rats. Furthermore, histopathological studies revealed cellular alterations in the myocardium. On the contrary, treatment with BSB has been observed to preserve the myocardium and restore all the cellular, molecular, and structural perturbations in the heart tissues of DOX-induced cardiotoxicity in rats. Results of the present study clearly demonstrate the protective role of BSB against DOX-induced cardiotoxicity, which is attributed to its potent antioxidant, anti-inflammatory, and antihyperlipidemic effects resulting from favorable modulation of numerous cellular signaling regulatory pathways, viz., Nrf2/Keap-1/HO-1, Akt/mTOR/GSK-3β, NF-κB/p38/MAPK, and NLRP3 inflammasomes, in countering the cascades of oxidative stress and inflammation. The observations suggest that BSB can be a promising agent or an adjuvant to limit the cardiac injury caused by DOX. Further studies including the role in tumor-bearing animals as well as regulatory toxicology are suggested.

## 1. Introduction

Cancer chemotherapy has advanced well and increases the survival and prognosis in survivors once recognized and treated in the early stages. Among numerous classes of available chemotherapeutic agents, the anthracycline class of cytotoxic drugs has been reputed for its use in the treatment of cancer for many decades [1]. In anthracycline antineoplastic agents, doxorubicin (DOX) is one of the popular agents, with a wide spectrum of use against various malignancies including lymphoma, leukemia, sarcoma, and carcinoma across all ages. It has become one of the cornerstones of chemotherapeutic regimens due to its high potency [2]. Despite the resistance of cancer cells and tumor recurrence, one of the major challenges with chemotherapeutic agents including DOX is the appearance of the adverse effects that adversely affect organ function and limit the clinical usage for chemotherapy [3]. One of the most prominent adverse effects of DOX is its effect on the heart, which leads to cardiotoxicity, which can be either acute or chronic following DOX treatment [4]. The appearance of acute cardiotoxicity following DOX treatment remains a serious concern for its use in chemotherapy [5]. Notably, in the past few years, numerous novel formulations of DOX or agents that can be used as an adjuvant or preventive with DOX have been the focus of research to minimize the adverse effects on the heart. 

The pathogenesis of cardiotoxicity has been studied extensively to develop novel pharmacological agents targeting the pathogenic events instituted by DOX [6]. Numerous mechanisms have been reported in the causation of DOX-induced cardiotoxicity, including erroneous deoxyribonucleic acid (DNA) repair, enhanced DNA damage, inhibition of topoisomerase-II, and cell replication, which culminates in irreversible injury to the heart and sequelae following oxidative stress, inflammation, and apoptotic cell death [7,8]. Life-threatening cardiotoxicity is one of the United States Food and Drug Administration (USFDA) black box warnings regarding the clinical usage of DOX, which limits therapeutic application of the drug [9]. As stated before, in addition to developing novel dosage forms, another approach is to explore agents that may have preventive or therapeutic effects for anticipated cardiotoxicity after DOX administration. The search for an effective and safe remedy to counter DOX-induced cardiotoxicity remains a critical issue [10]. 

To tackle DOX-induced cardiotoxicity, numerous agents possessing antioxidant, anti-inflammatory, and anti-apoptotic properties have been evaluated for their potential to provide cardioprotection against DOX chemotherapy [11]. The occurrence of overt generation of reactive oxygen species (ROS) and reactive nitrogen species (RNS), and activation of nicotinamide adenine dinucleotide phosphate NADPH oxidases (NOXs) and nitric oxide synthetases (NOSs), leads to an imbalance between oxidants and endogenous antioxidant defense, a cardinal feature of oxidative stress, and represents a major concern in DOX-induced cardiac injury [12]. The NADPH oxidase complex and its primary isoforms (NOX2 and 4) are among the important sources of free radicals, including ROS, in the heart [13]. NOX enzyme isoforms and their activation have been shown to play a crucial role in myocardial injury following DOX therapy [14,15]. The endogenous antioxidants such as glutathione (GSH), superoxide dismutase (SOD), and catalase get depleted when removing the free radicals triggered by DOX [16].

At the cellular level, another key regulator of oxidative stress is the nuclear factor erythroid 2-related factor 2 (Nrf2), a member of the Cap ‘n’ Collar family of basic leucine zipper proteins, which encode various antioxidant genes and detoxifying enzymes, and are well known to orchestrate redox defense systems during oxidative stress [17]. Nrf2 triggers transcription of Nrf2-associated antioxidant genes after being dissociated from Kelch-like ECH-associated protein 1 (Keap1) [18]. The expression of Nrf2 has been reported in DOX-induced cardiotoxicity, and increased activity and expression of Nrf2 have been shown to attenuate DOX-induced cardiotoxicity [19]. Moreover, inhibition of nuclear export/degradation of Nrf2 following alteration in the activation of Akt, a key player in cell survival, has been shown to limit oxidative stress in DOX-induced cardiotoxicity [20,21,22]. Akt activation has been further shown to prevent apoptosis of cardiomyocytes, while its inhibition reported to aggravate apoptosis and functional impairment in DOX-induced cardiotoxicity [23]. 

Cell survival signaling pathways, including Akt and glycogen synthase kinase-3 beta (GSK-3β), are well known to regulate myocardial cell survival, and thus appear as an important therapeutic target in modulating cell survival and death. Notably, DOX has been reported to induce a remarkable reduction in Akt and GSK3β phosphorylation in mouse hearts [21]. Additionally, the mammalian target of rapamycin (mTOR), an important and classical downstream mediator involved in the Akt signaling pathway, has been shown to play role in DOX-induced myocardial apoptosis by inhibiting the Akt/mTOR survival pathways [23]. Recent studies showed that mTOR inhibition contributes to the changes in cardiac mass and dysfunction, and its overexpression protected against DOX-induced cardiotoxicity [24]. 

Subsequent to oxidative stress, DOX also induces inflammatory cascades by triggering the release of proinflammatory cytokines (tumor necrosis factor-alpha (TNFα), interleukin-1beta (IL-1β), and interleukin-6 (IL-6)) following expression of inflammatory mediators (iNOS and COX-2), nuclear transcription factor kappa B (NF-κB), and mitogen-activated protein kinase (MAPK) signaling cascades in the myocardium [25]. Further, another cell signaling pathway that regulates inflammation is the NLR-family pyrin domain containing-3 protein (NLRP3) inflammasome, a core protein assembly (NLRP3, ASC, and caspase-1) that is one of the primary initiators of proinflammatory-mediated programmed cell death named pyroptosis by elevating the expression of interleukin-1beta (IL-1β) and interleukin-18 (IL-18). The activation of NLRP3 inflammasomes has been shown to participate in the onset and progression of DOX-induced cardiotoxicity [26]. The inhibition of NLRP3 inflammasomes has been shown to attenuate DOX-induced cardiac injury [27]. Recent reports also reveal that NLRP3 inflammasome activation is negatively regulated by Nrf2 and regulating the Nrf2-NLRP3 pathway represents a therapeutic target [28].

Since the recognition of DOX-induced cardiotoxicity, a large number of agents that possess antioxidant, anti-inflammatory, and anti-apoptotic properties have been evaluated in an experimental model of DOX-induced cardiotoxicity [11]. In recent years, enormous attention has been focused on exploring the protective effects of phytochemicals, plant-derived bioactive agents, possessing antioxidant, anti-inflammatory, and anti-apoptotic properties that may mitigate DOX-induced cardiotoxicity [29]. Among numerous available phytochemicals, the ones which are dietary in nature and have time-tested evidence of human usage and safety, and showed chemopreventive properties, are of notable importance for evaluation in DOX-induced cardiotoxicity [30]. 

The present study evaluated one such phytochemical, α-Bisabolol (BSB), in a rat model of DOX-induced cardiotoxicity. α-Bisabolol is one of the common ingredients in food, beverages, health supplements, edible preparations, and cosmetic products due to its floral aroma and ability to improve health and wellness [31]. BSB, chemically a natural monocyclic sesquiterpene alcohol also known as levomenol, is recognized by its nutty fruit aroma. It is abundantly present in a variety of plants, including German chamomile (*Chamomilla recutita* L.) [32], salvia (*Salvia runcinata*) [33], a flowering plant of the Myrtaceae family native to Panama *(Plinia Cerrocampanensi* Barrie) [34], and the wood of Candeia (*Eremanthus erythropappus*) [35]. 

α-Bisabolol is widely available, and dietarily bioavailable, possesses high lipophilicity, as well as exhibits numerous pharmacological properties including anti-inflammatory, wound healing, antibiotic, antimutagenic, antitumor, gastroprotective, analgesic, antimicrobial, and antioxidant [36]. α-Bisabolol gained popularity since its recognition by the United States Food and Drug Administration (USFDA) as ‘generally recognized as safe (GRAS) for human use and consumption. α-Bisabolol appears safe in experimental animals upon oral administration as its LD_50_ is in the range of 13–14 g/kg body weight [37]. Recently, BSB has been proven to protect against isoproterenol-induced myocardial infarction in rats [38,39,40]. α-Bisabolol has also been shown to exert protective effects in DOX-induced renal toxicity [41] and testicular toxicity [42], as well as anticancer and chemopreventive properties [43].

Considering the protective properties of BSB against different organ toxicities caused by DOX chemotherapy, it is worthwhile to investigate the efficacy of BSB in DOX-induced cardiotoxicity. Therefore, the present study aimed to investigate the cardioprotective effects of BSB against DOX-induced acute cardiotoxicity in rats. Further, to elucidate the underlying mechanisms, the cellular signaling pathways regulating oxidative stress, inflammation, and apoptosis were evaluated in a comprehensive manner. 

## 2. Results

### 2.1. BSB Maintained Body Weight, Reinstated Hemodynamics, and Preserved Cardiac Enzymes

**Figure 1:**
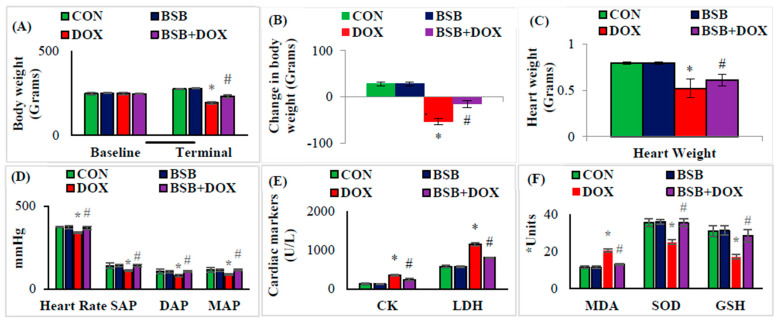
Effect of BSB on body weight (**A**), change in body weight (**B**), heart weight (**C**), hemodynamics (**D**), cardiac diagnostic markers (**E**), and oxidative stress markers (**F**). Each column is mean ± SD for eight rats in each group; columns not sharing a common symbol (*, ^#^) differ significantly from each other (* *p* < 0.05 vs. normal control, ^#^ *p* < 0.05 vs. DOX control). CON: Normal control rats; BSB: α-Bisabolol-alone treated rats; DOX: DOX-alone treated rats; BSB + DOX: α-Bisabolol-and-DOX-treated rats. * Units: µM/mL for MDA and GSH; U/mL for SOD.

The effect of BSB on body weight, hemodynamics, cardiac diagnostic enzymes, and oxidative stress markers are presented in Figure 1A–E. DOX injection to the rats caused a significant (*p* < 0.05) decrease in the body weight (Figure 1A), change in body weight (Figure 1B), and heart weight (Figure 1C) when compared to the normal control group, whereas BSB treatment significantly (*p* < 0.05) restored changes in body weight (Figure 1A,B) and heart weight (Figure 1C). DOX injection also caused a significant (*p* < 0.05) fall in the HR, MAP, SP, and DP (Figure 1D) in comparison with normal control rats. However, BSB treatment to DOX-injected rats produced significant (*p* < 0.05) restoration of hemodynamics (Figure 1D) in comparison with DOX control rats. As depicted in Figure 1E, DOX also induced a remarkable rise in cardiospecific diagnostic markers CK and LDH, and rise in these marker levels were significantly (*p* < 0.05) reversed by BSB treatment to near normal levels when compared to the DOX control group.

### 2.2. BSB Attenuated DOX-Induced Cardiac Oxidative Stress

**Figure 2:**
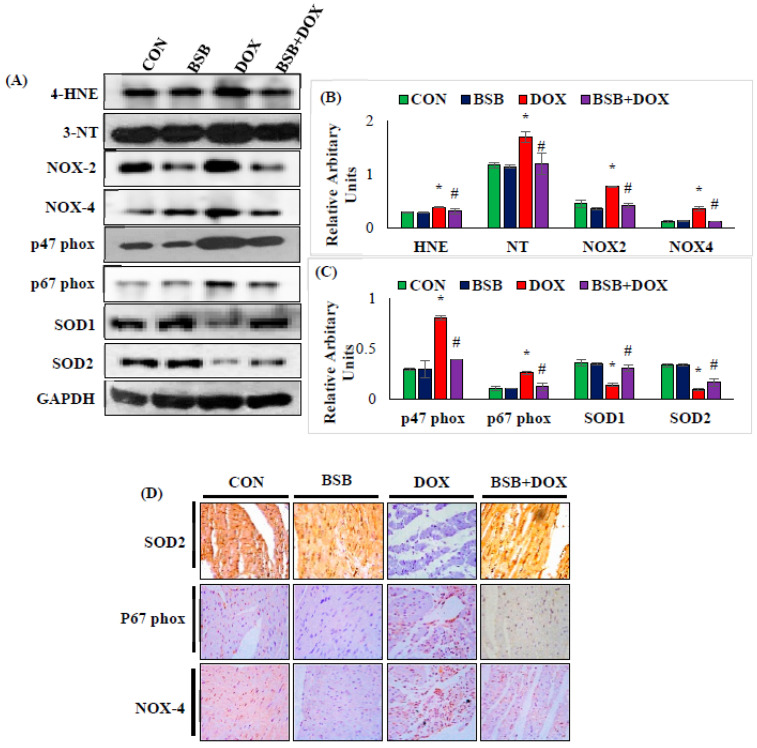
Immunoblotting of oxidative stress markers (**A**), densitometric analysis and changes in expression of HNE, 3-NT, NOX2 and 4, p47 phox, p67 phox, SOD1 and 2 (**B**,**C**), immunohistochemical analysis of SOD2, p67 phox and NOX4, magnification (40×) (**D**). Columns not sharing a common symbol (*, **^#^**) differ significantly from each other (* *p* < 0.05 vs. normal control, ^#^ *p* < 0.05 vs. DOX control). Immunoblotting and immunohistochemical analysis were performed in duplicates. CON: Normal control rats; BSB: α-Bisabolol-alone-treated rats; DOX: DOX-alone-treated rats; BSB + DOX: α-Bisabolol-and-DOX-treated-rats.

The effect of BSB on oxidative stress markers is presented in Figure 1F and Figure 2A–D. DOX injection induced a significant (*p* < 0.05) rise in the concentration of MDA along with a significant (*p* < 0.05) reduction in the activities of SOD and GSH levels when compared to the normal group (Figure 1F). BSB treatment significantly (*p* < 0.05) reversed the DOX-induced changes in MDA and SOD (Figure 1F). In addition, DOX injection to the rats caused a significant (*p* < 0.05) decrease in the expression of SOD1 and SOD2 with a significant (*p* < 0.05) increase in the expression of 4-HNE, 3-NT, p47 phox, p67 phox, NOX2 and NOX4 (Figure 2A–C). Immunohistochemical analysis of heart tissues confirms the expression of SOD2, p67 phox, and NOX4, in comparison with the normal control group (Figure 2D). However, DOX-injected rats upon treatment with BSB exhibited a significant (*p* < 0.05) increase in the myocardial expression of SOD1 and SOD2, along with a significant (*p* < 0.05) decrease in the expression of 4-HNE, 3-NT, p67 phox, and NOX2 and 4, and according to immunohistochemical analysis of SOD2, p67 phox, and NOX4 (Figure 2D), in comparison with DOX control rats.

### 2.3. BSB Favourably Modulated Nrf2/HO-1/Keap1 and Akt/mTOR/GSK-3β Signaling, and Nrf2 Nuclear Translocation and Binding Activity

**Figure 3:**
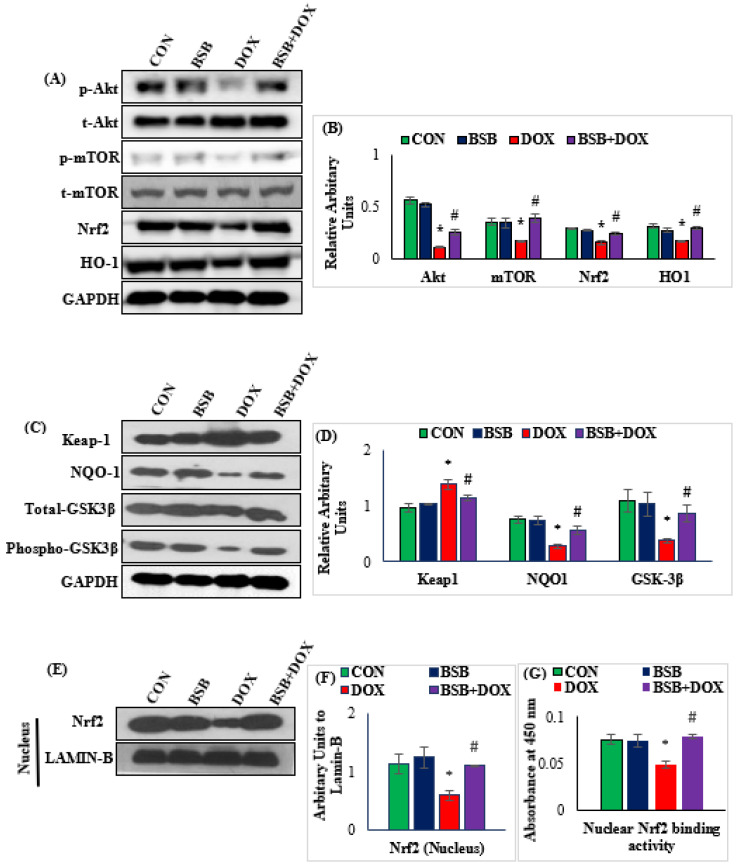
Western immunoblot analysis for Akt, mTOR, Nrf2, and HO-1 (**A**), densitometric analysis and relative changes in the expressions of Akt, mTOR, Nrf2, and HO-1 (**B**), Western immunoblot analysis for Keap1, NQO1, and GSK-3β signaling proteins (**C**), densitometric analysis and relative changes in the expressions of Keap1, NQO1, and GSK-3β proteins (**D**). Western immunoblot and densitometric analysis of Nrf2 in nuclear fraction depict nuclear translocation (**E**,**F**). Nuclear Nrf2 binding activity in different experimental groups (**G**). Each column is mean ± SD for eight rats in each group; columns not sharing a common symbol (*, ^#^) differ significantly from each other (* *p* < 0.05 vs. normal control, ^#^ *p* < 0.05 vs. DOX control). CON: Normal control rats; BSB: α-Bisabolol-alone-treated rats; DOX: DOX-alone-treated rats; BSB + DOX: α-Bisabolol-and-DOX-treated rats.

The effect of BSB on Nrf2/HO-1/Keap1 and Akt/mTOR signaling, and Keap1, NQO1, and GSK-3β and Nrf2 nuclear translocation and binding activity are presented in Figure 3A–G. DOX injection induced a significant (*p* < 0.05) fall in the expression of total cardiac and nuclear Nrf2, HO-1, phosphorylated Akt, mTOR, NQO1, and phosphorylated GSK-3β, and significantly (*p* < 0.05) increased Keap1 expression in rats when compared to the normal control group (Figure 3A–D). Moreover, a significant (*p* < 0.05) decrease in nuclear translocation of Nrf2 and Nrf2 binding activity was observed in DOX-injected rats compared to the normal control (Figure 3E–G). In contrast, treatment of DOX-injected rats with BSB significantly (*p* < 0.05) upregulated the expression of total cardiac and nuclear Nrf2, HO-1, phosphorylated Akt, mTOR, NQO1, and phosphorylated GSK-3β, and remarkably (*p* < 0.05) decreased Keap1 (Figure 3A–D), as well as significantly (*p* < 0.05) improving nuclear translocation of Nrf2 and Nrf2 binding activity (Figure 3E–G) in comparison with DOX control rats.

### 2.4. BSB Mitigated the Synthesis and Release of Proinflammatory Cytokines

**Figure 4:**
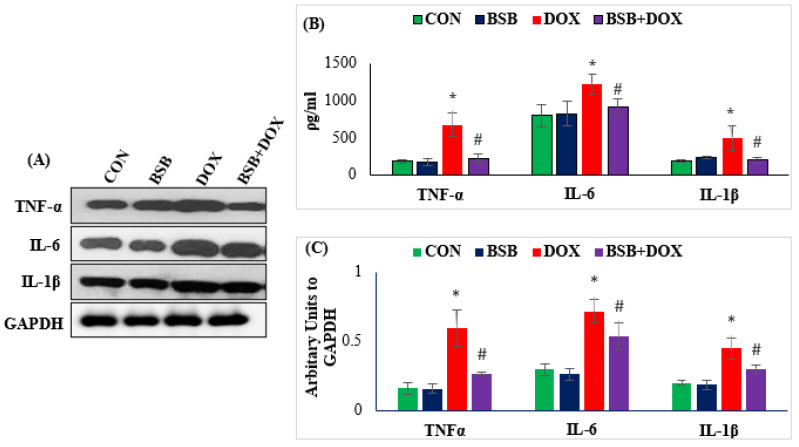
The levels of TNF-α, IL-6, and IL-1β in the myocardium (**A**), Western immunoblot analysis showing myocardial expression of TNF-α, IL-6, and IL-1β (**B**), densitometric analysis and relative changes in the expression of TNF-α, IL-6, and IL-1β (**C**). Each column is mean ± SD for eight rats in each group; columns not sharing a common symbol (*, ^#^) differ significantly from each other (* *p* < 0.05 vs. normal control, ^#^ *p* < 0.05 vs. DOX control). Immunoblotting analysis was performed in duplicates. CON: Normal control rats; BSB: α-Bisabolol-alone-treated rats; DOX: DOX-alone-treated rats; BSB + DOX: α-Bisabolol-and-DOX-treated rats.

The effects of BSB on the levels and expression of proinflammatory cytokines are presented in Figure 4A–C. The expression as well as the level of cytokines, IL-1β, IL-6, and TNF-α were significantly (*p* < 0.05) elevated in DOX-administered rats when compared to normal control rats (Figure 4A–C). In contrast, BSB treatment in DOX-injected rats produced a significant (*p* < 0.05) decrease in the levels and expressions of IL-1β, IL-6, and TNF-α when compared to DOX control rats (Figure 4A–C).

### 2.5. BSB Inhibited Inflammatory Mediators and Nuclear Factor Kappa B (NF-κB) Signaling Pathway Proteins

**Figure 5:**
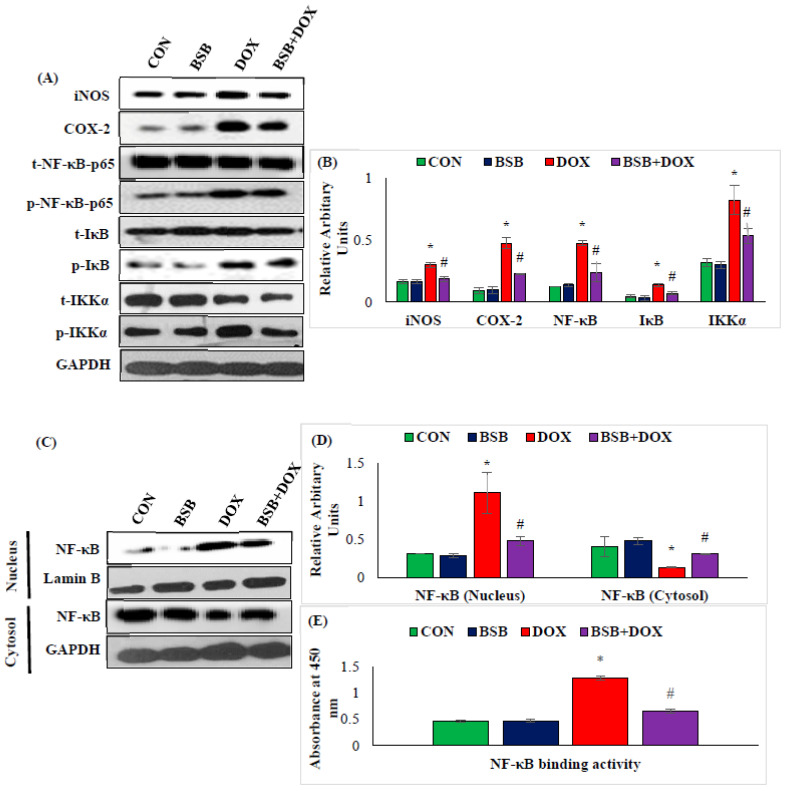
Western immunoblotting for iNOS, COX-2, NF-κB-p65, p-NF-κBp65, IκBα, p-IκBα, t-IKKα, and p-IKKα (**A**), relative changes in the expression of iNOS, COX-2, NF-κB, IκBα, and IKKα (**B**), Western immunoblotting analysis representing nuclear translocation of NF-κB (**C**), densitometric analysis and relative changes in the expression of NF-κB in nucleus and cytosol (**D**), NF-κB binding activity (**E**). Columns not sharing a common symbol (*, ^#^) differ significantly from each other (* *p* < 0.05 vs. normal control, ^#^ *p* < 0.05 vs. DOX control). Immunoblotting analysis was performed in duplicates. CON: Normal control rats; BSB: α-Bisabolol-alone-treated rats; DOX: DOX-alone-treated rats; BSB + DOX: α-Bisabolol-and-DOX-treated rats.

The effect of BSB on inflammatory mediators and the NF-κB signaling pathway are presented in Figure 5A–E. DOX-injected rats exhibited a significant (*p* < 0.05) rise in the myocardial expression of iNOS, COX-2, p-NFκB-p65, p-IκBα, and p-IKKα in comparison with normal control rats (Figure 5A,B). Moreover, a significant (*p* < 0.05) increase in the expressions/levels of NF-κB-p65 was observed in the nuclear fraction along with a significant (*p* < 0.05) decrease in the myocardial NF-κB-p65 expressions/levels in the cytosolic fraction and a significant (*p* < 0.05) increase in NF-κB binding activity in DOX-injected rats when compared to the normal control rats (Figure 5C–E). In contrast, DOX-injected rats treated with BSB exhibited a significant (*p* < 0.05) decrease in the expression of all these proteins when compared to DOX control rats (Figure 5A,B). Similarly, DOX-injected animals that received BSB treatment showed significant (*p* < 0.05) inhibition of NF-κB nuclear translocation and a significant (*p* < 0.05) decrease in NF-κB binding activity, when compared to normal control rats (Figure 5C–E).

### 2.6. BSB Inhibited MAPK Signaling Pathway

**Figure 6:**
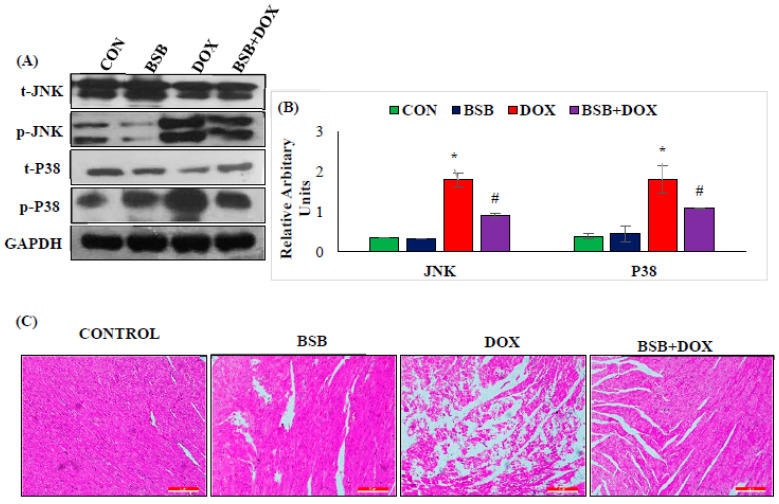
Immunoblotting analysis of t-JNK, p-JNK, P^38^, and p-P^38^ (**A**), densitometric analysis and relative changes in the expression of JNK and P^38^ (**B**). Columns not sharing a common symbol (*, **^#^**) differ significantly from each other (* *p* < 0.05 vs. normal control, ^#^ *p* < 0.05 vs. DOX control). The histopathology of the myocardium (10×) (**C**). Normal control rat’s heart revealed a regular architecture of the myocardium; BSB-alone-treated rat’s heart also showed normal intact muscle fibers without any pathological changes; DOX-administered rat’s heart showed extensive muscle fiber degradation with inflammatory cells; BSB-treated DOX-injected rats showed reduced muscle fiber degradation without inflammatory cells. Histological analysis was performed in duplicate. CON: Normal control rats; BSB: α-Bisabolol-alone-treated rats; DOX: DOX-alone-treated rats; BSB + DOX: α-Bisabolol-and-DOX-treated rats.

The effect of BSB on the MAPK signaling pathway is presented in Figure 6A,B. DOX-injected rats showed a significant (*p* < 0.05) increase in the expression of p-JNK and p-p38 signaling proteins without significant alterations in the total proteins of these isoforms (Figure 6A,B). In contrast, BSB treatment to DOX-injected rats produced a significant (*p* < 0.05) decrease in the phosphorylation of MAPK signaling proteins when compared to the DOX control rats (Figure 6A,B).

### 2.7. BSB Preserved Myocardial Histoarchitecture in DOX-Induced Cardiotoxicity

The histopathological changes in heart issues are displayed in Figure 6C. The optical microscopic examination of normal and BSB control (BSB per se or alone) rats exhibited noram myocardial histology, as illustrated by intact morphology and myofibrils. However, rats in the DOX control group showed disrupted myofibrils and separation of cardiac muscle fibers, necrosis, edema, and inflammatory cells. DOX-injected rats treated with BSB displayed the salvage of myocardium evidenced by the preservation of cellular changes, along with lesser inflammation, edema, cell death, and occasional myofibrillar damage. 

### 2.8. BSB Ameliorated Alterations in the Lipids Levels in the Serum

**Figure 7:**
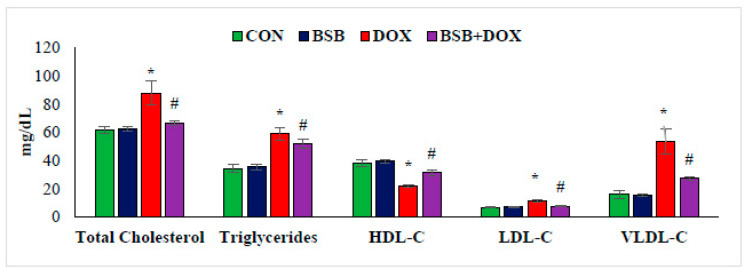
The levels of lipids in the serum. Each column is mean ± SD for eight rats in each group; columns not sharing a common symbol (*, ^#^) differ significantly from each other (* *p* < 0.05 vs. normal control, ^#^ *p* < 0.05 vs. DOX control). CON: Normal control rats; BSB: α-Bisabolol-alone-treated rats; DOX: DOX-alone-treated rats; BSB + DOX: α-Bisabolol-and-DOX-treated rats.

The effect of BSB on lipid profile is presented in Figure 7. The levels of total cholesterol, triglycerides, LDL, and VLDL were significantly (*p* < 0.05) increased concomitant to a significant (*p* < 0.05) decrease in HDL cholesterol in DOX-injected rats, when compared to normal control rats (Figure 7). However, DOX-administered rats treated with BSB showed a significant (*p* < 0.05) correction in the serum levels of lipids and lipoprotein when compared to DOX control rats (Figure 7).

### 2.9. BSB Reduced NLRP3 Inflammasome Activation-Mediated Pyroptosis in Rats

**Figure 8:**
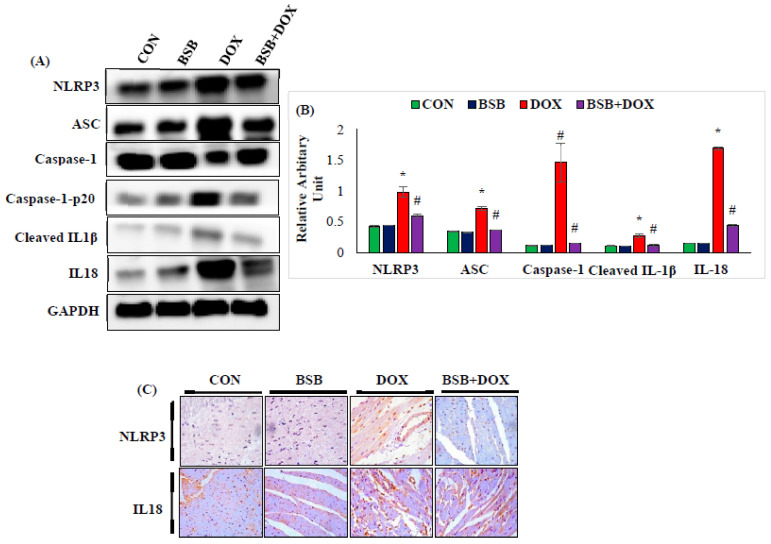
Immunoblotting and densitometric analysis of pyroptosis marker proteins, NLRP3, ASC, caspase-1, caspase-1-p20, cleaved IL-1β, and IL-18 (**A**), relative changes in the expression of NLRP3, ASC, caspase-1, caspase-1-p20, cleaved IL-1β, and IL-18 (**B**), immunohistochemistry of NLRP3 and IL-18, magnification (40×) (**C**). Columns not sharing a common symbol (*, ^#^) differ significantly from each other (* *p* < 0.05 vs. normal control, ^#^ *p* < 0.05 vs. DOX control). Immunoblotting and immunohistochemical analysis were performed in duplicate. CON: Normal control rats; BSB: α-Bisabolol-alone-treated rats; DOX: DOX-alone-treated rats; BSB + DOX: α-Bisabolol-and-DOX-treated rats.

The effect of BSB on the NLRP3 inflammasome pathway is presented in Figure 8A–C. DOX injection into rats showed significant (*p* < 0.05) increase in the myocardial protein expression of components of NLRP3 inflammasome representing pyroptosis proteins, as depicted by both Western blotting (NLRP3, ASC, caspase-1-p20, cleaved IL-1β, IL-18) and immunohistochemistry (NLRP3 and IL-18) (Figure 8A–C) in comparison with normal control rats. However, DOX-injected rats treated with BSB produced significant (*p* < 0.05) reduced expression of these signaling proteins when compared to DOX control rats.

## 3. Discussion

The present study is the first to demonstrate the protective effect of BSB in DOX-induced acute cardiotoxicity in rats and elucidated the underlying mechanism by favorable modulation of various cell signaling regulatory mechanisms, viz., Nrf2/Keap-1/HO-1 and Akt/mTOR/GSK-3β, NF-κB/p38/MAPK, and NLRP3 inflammasome pathways in countering the cascades of oxidative stress and inflammation.

In the present study, DOX administration caused weight loss that can be reasonably ascribed to impaired gastrointestinal function and reduced food intake followed by nutrient malabsorption [44]. The restoration of the body weight following BSB treatment can be considered a positive sign that is suggestive of the beneficial effects of BSB in improving general health and well-being against DOX-induced cardiotoxicity in rats. DOX injection in rats also showed reduction in the heart weight, which could be related to myocardial cell death either by necrosis or apoptosis. However, the restoration of heart weight following BSB treatment reveals that BSB has the potential to protect the heart from the deleterious effects of DOX in rats [45]. In addition to alterations in heart and body weight, DOX injection also caused a significant rise in the serum levels of cardiac diagnostic markers such as CK and LDH, which have been considered standard diagnostic indicators of cardiac injury and display cardiotoxicity in rats [46]. BSB-treatment to DOX-injected rats showed significant restoration of diagnostic markers of cardiac injury, which demonstrates reduced leakage of these enzymes due to maintenance of cellular permeability and is suggestive of the membrane-stabilizing and preserving property of BSB. 

Further, myocardial membrane lipid peroxidation evidenced by increased formation of MDA supports the observation of DOX-induced overt generation of free radicals that leads to the destabilization of membranes and the release of cardiac enzymes in the serum [26]. The ability of BSB to preserve the membrane and protect the integrity of the myocardium indicates the liberation of cardiac enzyme injury markers in circulation. In addition to the cardiospecific markers, the important parameters of cardiac function are the hemodynamic parameters. DOX administration caused deterioration in the cardiac functions, followed by impaired diastolic and systolic functions of the myocardium [47]. BSB treatment was observed to positively restore the altered hemodynamics in DOX-administered rats and revealed the ability of BSB to correct hemodynamic alterations and improve cardiac function. 

The semiquinones formed in metabolism of DOX, which is a short-lived and highly toxic metabolite, interacts with molecular oxygen and initiates the chain of reactions leading to the production of hydroxyl radicals and superoxide anions [47]. DOX increases the susceptibility of the cardiomyocytes to ROS and RNS by lessening enzymatic (SOD) and non-enzymatic antioxidant (GSH) status. These endogenous antioxidants defense is involved in the clearing of ROS by utilizing hydroxyl radicals and superoxide anions [48]. The declined antioxidant status following DOX injection has been attributed to their utilization in scavenging ROS [49]. In normal cells, more than 90% of glutathione is in its reduced form; GSH. Glutathione, a low-molecular-weight non-protein thiol, as an endogenous antioxidant, regulates physiological levels of ROS to maintain cellular homeostasis and imparts resistance to oxidative stress induced by xenobiotics-including chemotherapeutic drugs [50]. DOX triggers the depletion of the cellular content of GSH concomitant to the increased lipid peroxidation, and BSB treatment in the present study was observed to prevent the depletion of GSH with an inhibition of MDA formation, which demonstrates its antioxidant property. 

Enhanced oxidant and electrophile generation during DOX chemotherapy triggers myocardial membrane lipid oxidation, resulting in the buildup of a potent reactive electrophile named 4-HNE, which possesses the ability to alter the cardiac function and is, in particular, a standard marker of DOX-induced oxidative stress [51]. NADPH oxidases (NOX2 and 4 isoforms) are crucial enzymatic sources involved in ROS formation, which mainly contributes to the induction of cardiomyocyte apoptosis [52]. 3-NT, a standard marker indicating the formation of peroxynitrite and nitrative stress, is the primary downstream effector promoting DOX-induced cardiotoxicity [53]. 

Further, the NADPH oxidase enzyme complex abundantly present in the mitochondrial membrane is the source of ROS, as it acts as an electron donor to oxygen and generates oxygen free radicals and interferes with redox signaling. NADPH oxidase is one of the main targets for the conversion of DOX to semiquinone, which leads to ultrastructural injuries to mitochondria and sarcoplasmic reticulum [54]. The NADPH oxidase complex consists of a catalytic NOX2 to which several cytosolic subunits, such as p47phox and p67phox, get associated in the activated enzyme. Inhibition of NOX2 and 4, as well as p47phox and p67, has been reported to prevent oxidative stress, a common reason to cardiac injury [54]. 

Additionally, the antioxidant enzyme, SOD1, a copper/zinc enzyme isoform abundantly present in the cytoplasm, converts superoxide to hydrogen peroxide and molecular oxygen. By comparison, the SOD2 isoform catalyzes the dismutation of O_2_^·−^ produced by the electron transport chain to H_2_O_2_, with the generation of O_2_ in both the mitochondrial matrix and intermembrane component, and plays an important role in the detoxification of the mitochondrial ROS [55]. The myocardial expression of the antioxidant enzymes SOD1 and SOD2 was decreased in DOX-injected rats; however, this decrease was significantly reversed by BSB treatment. 

Consistent with previous reports, the present study also showed increased 4-HNE, 3-NT, p47 phox, p67 phox, NOX2, and NOX4 expression, as well as decreased expression of SOD1 and 2, ascribed to the overproduction of ROS and RNS subsequent to DOX administration [53,56]. The alterations in expression of SOD2, p67phox, and NOX4 were further confirmed by immunohistochemistry. However, treatment with BSB produced a remarkable reduction in the levels of 4-HNE, 3-NT, p47 phox, p67 phox, NOX2, and NOX4 expression, as well as showing improvement in the expression of SOD1 and 2, along with the reversal of changes in SOD2, p67phox, and NOX4 induced by DOX in cardiomyocytes. In the present study, BSB treatment was observed to attenuate oxidative and nitrative stress and lipid peroxidation by improving antioxidant enzymes, inhibiting NOX isoforms actvities, reducing oxidative stress mediators, thus imparted protective effects against DOX-induced cardiotoxicity.

Other cellular signaling mediators of antioxidant defense are Nrf2, Keap1, and HO-1, which have been shown associated with the incidence of DOX-induced oxidative damage and apoptosis in cardiac cells [57]. Nrf2, a key transcription factor in normal conditions, exists with Kelch-like ECH-associated protein 1 (Keap1), and in the event of oxidative stress, Nrf2 is dissociated from Keap1 and binds to the elements of antioxidant responses, which further results in the transcription of Nrf2-associated antioxidant genes, including HO-1 [18]. HO-1 and NQO1, generated in response to oxidative stress, are the downstream factors of Nrf2 activation and are considered important intracellular antioxidants against intracellular and extracellular oxidative stress [58]. Further, NAD(P)H: quinone acceptor oxidoreductases (NQOs) are present in many isoforms, mainly NQO1, which is inducible, and play a role in reducing endogenous and exogenous quinones to hydroquinones. The myocardial expression and activities of Nrf2/HO-1 are shown to decrease in DOX-induced injury to the heart with a concomitant increase in Keap1 expression [23]. Consistent with previous reports, the present study also demonstrates significant upregulation of Keap1 with a reduction in expression of Nrf2/HO-1 and NQO1 proteins after DOX administration [59,60]. However, BSB treatment markedly improved the myocardial expression of Nrf2/HO-1 and NQO1 proteins and decreased the expression of Keap1. The favorable changes in Nrf2/Keap1/HO-1 and NQO1 signaling further demonstrate the antioxidant activity of BSB and its role in providing protection against DOX-induced cardiac injury. 

Further, glycogen synthase kinase-3 β (GSK-3 β), a redox-sensitive multifunctional serine/threonine protein kinase, is well known to regulate apoptosis as well as oxidative stress by targeting Nrf2 in cardiomyocytes, and its inhibition has been shown to provide protection against DOX-induced cardiac injury. GSK-3β has been shown to phosphorylate several upstream and downstream components of the AKT/mTOR signaling network [21]. Akt is the key signal transduction protein that phosphorylates several substrates and downstream effectors, including mTOR, and Akt/mTOR inactivation has also been shown to play a role in DOX-induced cardiotoxicity, whereas enhanced phosphorylation of Akt has been reported to prevent DOX-induced cardiotoxicity [61,62]. Akt inactivation also influences the expression of GSK-3β by triggering myocardial Nrf2 nuclear elimination and its cytoplasmic degradation in DOX-injected rats [63]. Consistent with previous studies [63], DOX triggered a reduction in the expression of phosphorylated AKT and phosphorylated GSK-3β, and mTOR, whereas BSB treatment reversed the expression of these proteins. The observations of the present study clearly reveal that BSB renders protective effects against oxidative stress and eventually cell death due to its potential to activate GSK-3β/Akt/mTOR signaling, which is in line with previous studies [63].

In addition to invoking oxidative stress, DOX also triggers inflammatory events in the myocardium by the enhanced release of proinflammatory cytokines (IL-1β, TNF-α, and IL-6), induction of the inflammatory mediators (COX-2 and iNOS), and perturbations in NF-κB signaling, which are well-known players in the acute and chronic inflammatory process [64,65]. NFκB p65, a crucial proinflammatory transcriptional factor activated in response to various stimuli including ROS upon activation and nuclear translocation, may trigger inflammatory response and apoptosis [54]. The downstream regulators of NF-κB p65, such as COX-2 and TNF-α, showed an aggravated response to DOX administration. Upon stimulation by oxidative stress or proinflammatory cytokines, IκBα is phosphorylated by its upstream IκB kinase (IKK), triggering IκB polyubiquitination and degradation by proteases, thereby causing the nuclear translocation of NF-κB [66]. NF-κB activation augments nitric oxide by inducing the expression of iNOS [67]. The findings of the present study showed DOX-induced NF-κB phosphorylation, activation, and nuclear translocation in the myocardium. Notably, BSB treatment observed to inhibit phosphorylation of IKKα, NF-κB-p65, and IκBα in the myocardium, that reveal activation and translocation of NF-κB were reduced in BSB-treated rats.

Furthermore, MAPKs, serine/threonine protein kinases, control cell differentiation, proliferation, survival, and death, along with their key role in controlling inflammatory mediators. They also get activated in response to ROS-induced oxidative and nitrative stress, leading to the enhancement of apoptosis [53,68]. MAPK family proteins (JNK and p38) also showed a considerably increased MAPK activation in DOX-induced cardiac injury, in addition to a rise in the levels/expressions of the cytokines and inflammatory mediators. However, BSB treatment administered to DOX-injected rats inhibited DOX-induced NF-κB activation and significantly inhibited the phosphorylation of MAPK signaling proteins, along with attenuating inflammatory mediators. This displays the positive regulation of MAPK family proteins, JNK and P^38^, and reasonably explains the underlying anti-inflammatory mechanisms of BSB in DOX-induced cardiotoxicity. 

Recent studies have suggested that the NLRP3-triggered inflammatory responses and pyroptosis play key role in DOX-induced cardiotoxicity [69]. Pyroptosis is an important natural immune response depending on activated caspase-1 and is associated with distortion to cell integrity, cell swelling, and the release of intracellular proinflammatory cytokines, that leads to the progression of cardiovascular diseases [70]. NLRP3 inflammasomes induce pyroptosis, accompanied by caspase-1 activation. Doxorubicin is well known to augment the activity of myocardial NLRP3 inflammasome following the enhanced release of IL-1β and increased activity of caspase-1, which leads to the development of oxidative and inflammatory events and myocardial apoptosis, eventually resulting in cardiac dysfunction [71]. Inhibiting myocardial apoptosis and NLRP3 is considered an effective cardioprotective strategy and has also been reported to ameliorate DOX-induced cardiotoxicity [71]. DOX administration significantly augmented the expression of NLRP3, ASC, caspase-1-p20, cleaved IL-1β, and IL-18 proteins, consistent with previous studies [69]. The present study findings show the ability of BSB to mitigate NLRP3 inflammasome activation-mediated pyroptosis by reducing the expression of NLRP3, ASC, cleaved caspase-1-p20, cleaved IL-1β, and IL-18, in DOX-injected rats. As discussed earlier, BSB has been also shown to upregulate the Nrf2 signaling pathway in DOX-induced cardiotoxicity. Taken together, the observations of the present study findings demonstrate that the protective effect of BSB on DOX-induced cardiac injury involves the favourable modulation of the Nrf2-NLRP3 signaling pathway. 

Apart from oxidative stress and inflammation, DOX also triggers an alteration in the lipid profile of the rats [72]. Hyperlipidemia is one of the major pathological alterations that promotes negative effects on cardiac function and partly contributes to the pathogenesis of heart diseases [73]. Consistent with previous reports, our study also displayed increased levels of total cholesterol, triglycerides, LDL, and VLDL, along with decreased HDL cholesterol in DOX-injected rats [74]. However, BSB treatment remarkably corrected the serum levels of lipids and lipoprotein, which clearly reveals its antihyperlipidemic property and that it imparts cardioprotective effects. 

The biochemical and molecular alterations in the myocardium following DOX administration were further supported by histopathological assessment in light microscopic examination. DOX administration induced clear morphological changes in cardiac tissues, as evidenced by remarkable morphological changes, i.e., cytoplasmic vacuolization, necrosis, edema, separation of myofibers, and myofibrillar disorganization, in agreement with numerous previous studies [75]. However, treatment with BSB salvages the cardiac cells, prevents the loss of myofibers, and preserves the histoarchitecture matching to the normal morphology. The reduced lipid peroxidation product and membrane stabilization supported by reduced leakage of cardiac enzymes reinforce the cardioprotective property of BSB on the histology of heart tissues. The appearance of an intact morphology and absence of histopathological features following only BSB treatment (BSB per se group) further demonstrate that BSB at the doses studied is effective as well as devoid of deleterious effects on heart tissues. 

## 4. Materials and Methods

### 4.1. Drugs and Chemicals

α-Bisabolol, duponol, doxorubicin, L-cysteine, α-ketoglutaric acid, xylenol orange, butylated hydroxytoluene, tri-sodium citrate, thiobarbituric acid, trichloro acetic acid, 2,4-dinitro phenyl hydrazine, oxaloacetate, hexokinase, and 1,1′,3,3′ tetra methoxy propane were obtained from Sigma Chemicals Co., St. Louis, MO, USA. All other chemicals used in the present study possess standards of analytical grade.

### 4.2. Experimental Animals

Adult male Wistar rats (weight 180–190 g), used in the present study were housed at the animal research facility of the College of Medicine and Health Sciences, United Arab Emirates University (UAEU), Al Ain, United Arab Emirates. The animals were acclimatized and maintained at standard animal house conditions. A maximum of three rats were kept in animal cages and fed a chow diet and water ad libitum. The animal ethics protocol was approved by the Institutional Animal Ethics Committee of UAE University, UAE. 

### 4.3. Induction of Cardiotoxicity in Wistar Rats

An intraperitoneal (i.p.) injection of DOX (12.5 mg/kg, body weight) was given to male Wistar albino rats for the induction of acute cardiotoxicity following our previous studies, including our laboratory studies [44]. The dose of the BSB was chosen based on a previous study [43]. The confirmation of DOX-induced cardiotoxicity in rats was established by changes in the levels of cardiospecific diagnostic enzyme markers, including creatine kinase (CK) and lactate dehydrogenase (LDH) in the serum.

### 4.4. Experimental Design

The experimental animals were randomly separated into four experimental groups, each containing fifteen rats. The rats in group I were allocated to the normal control and received vehicle only. The rats in group II were allocated to the BSB group and received BSB (25 mg/kg body weight, p.o. twice daily) for five days. The rats in group III were allocated to the DOX group given a single intraperitoneal injection of DOX (12.5 mg/kg body weight), and group IV (DOX + BSB) were given an intraperitoneal injection of DOX (12.5 mg/kg body weight) and administered BSB (25 mg/kg body weight, p.o. twice daily) for five days. BSB was administered on the same day as the DOX treatment and post-treated until sacrifice. Olive oil was used as a vehicle and administered to all the study groups in a similar volume. Body weight and hemodynamics were recorded before and after treatment. On day 6, the animals were sacrificed, and blood was collected to separate serum and stored at −80 °C. The heart tissues were isolated, weighed, and snap frozen in aluminum foil, kept in liquid nitrogen, and further stored at −80 °C, for biochemical analysis and immunoblotting assays. The tissues for the histological study were fixed in 10% neutralized buffered formalin and stored at 4 °C.

### 4.5. Assay of the Cardiac Marker Enzymes

The serum concentrations of CK and LDH were determined using a veterinary autoanalyzer (Vet Test 8008 Chemistry Analyzer, West Yorkshire, UK).

### 4.6. Hemodynamic Assessments

The hemodynamic parameters, viz., heart rate (HR), systolic, mean arterial, and diastolic blood pressure (SP, MAP, and DP) were recorded by the tail-cuff method employing Kent CODA, a noninvasive blood pressure measuring system (Kent Scientific Corporation., Torrington, CT, USA). 

### 4.7. Estimation of Lipid Peroxidation and Antioxidants

The occurrence of lipid peroxidation was measured by malondialdehyde (MDA) concentration in the heart tissues employing a commercially available kit (Northwest life sciences, Vancouver, WA, USA). The activities/concentrations of SOD and GSH in the whole heart were estimated using the procedure provided with the commercial kits (Cayman Chemicals Co., Ann Arbor, MI, USA and Sigma-Aldrich Co., St. Louis, MO, USA).

### 4.8. Enzyme Linked Immunosorbent Assay (ELISA)

The concentrations of interleukin 6 (IL-6), interleukin-1βeta (IL-1β), and tumor necrosis factor alpha (TNF-α) in the heart tissue homogenates were determined using the available commercial ELISA kits (R & D Systems, Minneapolis, MN, USA). 

### 4.9. Estimation of Lipids and Lipoproteins in the Serum

The estimations of serum total cholesterol, triglycerides (TGs), high-density lipoprotein cholesterol (HDL-c), low-density lipoprotein cholesterol (LDL-c), and very-low-density lipoprotein cholesterol (VLDL-c) were performed using available commercial kits (Abcam, Waltham, MA, USA). 

### 4.10. Isolation of Heart Nuclear and Cytosolic Fractions

Myocardial subcellular fractions (nuclear, and cytosolic) were isolated employing available commercial kits (Abcam, Waltham, MA, USA).

### 4.11. Transcription Factor Binding Assay (NF-κBp65 Binding Activity)

The heart tissues were subjected to nuclear/cytoplasmic fractionation as mentioned in the protocol (Abcam, Waltham, MA, USA). The isolated cardiac nuclear extracts were assessed for NF-κB-p65 binding activity (Abcam, Waltham, MA, USA).

### 4.12. Nrf2 Transcription Factor Assay

Nrf2 transcription factor assay was performed using 20 µg of nuclear proteins by measuring nuclear Nrf2 binding activity at 450 nm following the manufacturer’s instructions available with the kit (Abcam, Waltham, MA, USA).

### 4.13. Western Blot Analysis

The heart tissues were weighed and homogenized in radio-immunoprecipitation assay (RIPA) extraction buffer (Sigma Aldrich Co., St. Louis, MO, USA) following the addition of protease and phosphatase inhibitors, and the homogenate was cold centrifuged for 30 min at 14,000 rpm. The supernatant obtained was mixed with Laemmlli buffer (4×) and 2-mercaptoethanol (Bio-Rad, CA, USA and Sigma Aldrich, St. Louis, MO, USA). An equal concentration of proteins was separated and transferred onto the polyvinylidene fluoride (PVDF) membranes (Thermo Fisher Scientific, Rockford, IL, USA) following the gel electrophoresis procedure. Further, the membrane was incubated overnight at 4 °C with primary antibodies against inducible nitric oxide synthase (iNOS), (anti-rabbit; Sigma Aldrich, MO, USA), 4-hydroxynonenal (4-HNE), 3-nitrotyrosine (3-NT), NADPH oxidase 2 and 4 (NOX2 and 4), cyclooxygenase-2 (COX-2), nuclear factor of kappa light polypeptide gene enhancer in B-cells inhibitor-alpha (IκB-α), p-IκB-α, toll like receptor 4 (TLR4), Nrf2, heme oxygenase-1 (HO-1), total p38 mitogen-activated protein kinases (t-P^38^), phosphorylated p38 mitogen-activated protein kinases (p-P^38^), phospho-extracellular signal-regulated kinase (p-ERK), NLRP3, Apoptosis-associated speck-like protein containing a caspase recruitment domain (ASC), IL-6, interleukin-18 (IL-18), procaspase-1, (anti-rabbit and mouse, Abcam, MA, USA), pro-IL1beta, phosphorylated-c-Jun N-terminal kinases (p-JNK), TNF-α, IL-1β, total c-Jun N-terminal kinases (t-JNK), (Santa Cruz Biotechnology, Dallas, TX, USA), total protein kinase B (t-Akt), phosphorylated protein kinase B (p-Akt), total mammalian target of rapamycin (t-mTOR), phospho mammalian target of rapamycin (p-mTOR), superoxide dismutase-1 (SOD1), superoxide dismutase-1 (SOD2), IKKα, phosphorylated IκB kinase alpha (p-IKKα), glyceraldehyde-3-phosphate dehydrogenase (GAPDH) (Cell signaling Technology, Danvers, MA, USA), NF-κB-p65, GAPDH, and p-NF-κB-p65 (anti-mouse; Millipore, USA). Further, the primary antibody processed membrane was washed with 1X washing buffer containing tris-buffered saline Tween 20 (TBST) three times and further subjected to incubation with corresponding secondary antibodies for one hour, and the protein expression was determined using chemiluminescence West pico and signal enhancer kits (Thermo Fisher Scientific, Rockford, IL, USA). The signal intensities were estimated by densitometric analysis using Image J software (ij144) (NIH, USA). 

### 4.14. Histopathological Evaluations

For histological analysis, heart tissue fragments were fixed in 10% formalin. The fixed tissues were dehydrated in serial dilutions of alcohol and xylene. Before embedding in paraffin The sections of 4–5 µm thickness were cut and stained with hematoxylin and eosin (H&E) to detect pathological changes. The stained slides were visualized under a light microscope and images were captured (10×) (BX41, Olympus, Japan).

### 4.15. Immunohistochemical Staining

To perform Immunohistochemical staining, 4–5 µm sections were collected on silane-coated microscope slides. Heat induced antigen retrieval was performed followed by incubating with peroxidase (3% hydrogen peroxide) blocking and application of primary antibodies. The mouse- and rabbit-specific horseradish peroxidase/3,3′-diaminobenzidine detection immunohistochemistry kit (Vector Laboratories, CA, USA) was used for development after primary antibody incubation followed counterstaining with hematoxylin.

### 4.16. Protein Estimations in the Heart

The estimation of protein content in the samples was performed following the instructions supplied with the commercially available bicinchoninic acid (Pierce^TM^ BCA protein) assay kit (Thermo Fisher Scientific, Rockford, IL, USA).

### 4.17. Statistical Analysis

The data are presented as the mean ± standard deviation (SD). The analysis of data was performed with a one-way analysis of variance (ANOVA) following Duncan’s Multiple Range Test (DMRT) using version 28 of IBM SPSS software. A *p*-value less than 0.05 was considered statistically significant. 

## 5. Conclusions

Taken together, the present findings demonstrate that BSB treatment protects against DOX-induced acute cardiotoxicity in rats by favorable modulation of various cell signaling regulatory mechanisms, viz., Nrf2/Keap-1/HO-1 and Akt/mTOR/GSK-3β, NF-κB/p38/MAPK, and NLRP3 inflammasome pathways, in countering the cascades of oxidative stress and inflammation. The findings suggest that either BSB or BSB-containing plants could be an effective alternative or adjunct to reduce or resist the adverse effects of chemotherapeutic-agent-induced cardiotoxicity. Corroborating the present study findings and the beneficial effects of BSB in cancer chemotherapy, observations of our study also may help in reducing the dose-dependent toxicity of DOX. The effects also may indicate the prospect of using BSB as an agent alone, as an adjuvant in reducing cardiotoxicity in adult and childhood cancer survivors, especially in those treated with higher doses of DOX, subject to regulatory toxicology and human studies. 

## Data Availability

This is an original research article, and the data originating in the study are presented in the manuscript.
